# The treatment of complex intra-articular distal radius fractures with turning radius and distal volaris radius plate fixation

**DOI:** 10.1186/s40001-020-00470-x

**Published:** 2020-12-07

**Authors:** Zhaofeng Jia, Shijin Wang, Wei Jiang, Chuangli Li, Jiandong Lin, Qisong Liu, Guangheng Li, Xinjia Hu

**Affiliations:** 1 Department of Osteoarthropathy and Institute of Orthopedic Research, Shenzhen People’s Hospital, The Second Clinical Medical College of Jinan University and the First Affiliated Hospital of Southern University of Science and Technology, Shenzhen, 518035 Guangdong China; 2 Department of Orthopaedics, Taian City Central Hospital, Taian, 271000 Shandong China

**Keywords:** Intra-articular distal radius fracture, Dorsal displaced, Fracture internal fixation, Distal volaris radius, Distal palmar approach, Radius turning

## Abstract

**Background:**

Although distal radius fractures (DRFs) are clinically common, intra-articular DRFs accompanied by dorsally displaced free fragments are much less so. At present, it is very difficult to fix and stabilize the intra-articular distal radius fractures accompanying dorsally displaced free fragments with a plate. Our aim was to investigate the clinical effect of DRFs with distally displaced dorsal free mass treated with distal volaris radius (DVR) combined with turning of the radius via the distal palmar approach.

**Methods:**

From 2015 to 2019, 25 patients with intra-articular distal radius fractures associated with dorsally displaced free fragments were selected and treated with distal volaris radius (DVR) combined with turning of the radius via the distal palmar approach. This study involved 14 males and 11 females, with an average age of 34.5 years (ranging from 21 to 50 years). The mean follow-up period was 16.5 months (ranging from 12 to 22 months). The dorsal displacement of the free fragments was analyzed by X-ray and three-dimensional computed tomography, allowing characterization of postoperative recovery effects by radial height, volar tilt and radial inclination. For the follow-up, we evaluated effects of the surgery by analyzing range of motion (ROM); Modified Mayo Wrist Score (MMWS); and Disabilities of Arm, Shoulder and Hand (DASH) score. Postoperative wound recovery and complications were also monitored to evaluate the clinical therapeutic effects of the surgical procedures.

**Results:**

X-ray showed that all patients showed reduced fractures, well-healed wounds and recovered function with no obvious complications. Based on the follow-up, patients had a mean radial height of 10.5 mm (ranging from 8.1 to 12.6 mm), mean MMWS of 78.8° (ranging from 61° to 90°), mean DASH score of 16.25 (ranging from 11 to 21), mean ROM for volar flexion of 76.5° (ranging from 62° to 81°), mean ROM for dorsiflexion of 77.1° (ranging from 59 to 83) and mean VAS score of 1.4 (ranging from 1 to 3).

**Conclusion:**

Treatment of the intra-articular distal radius fractures accompanying dorsally displaced free fragments with turning of the radius and the DVR plate system via the distal palmar approach is effective and has no obvious complications.

## Background

Distal radius fractures (DRFs) are common fractures in adults, usually caused by high-energy trauma [1]. High-energy injuries often lead to DRFs with dorsally or volarly displaced free fracture fragments; however, the incidence of intra-articular distal radius fractures accompanying dorsally displaced free fragments is very rare, making volarly locking plate fixation difficult. This kind of fracture belongs to the class of intra-articular fractures, which require anatomical reduction and firm fixation. If the fixation is not stable enough, the fracture may be displaced and even undergo subluxation, which may lead to malunion or non-union of the fracture, as well as early onset of traumatic arthritis and impaired wrist function. So far, no consensus has been reached on the definition of this type of injury, its mechanism or optimal treatment protocols [2–4]. Tendon irritation and even serious complications, including tendon rupture, can easily occur when the plate is fixed via the dorsal approach. The reduction and fixation effect of the palmar combined dorsal approach was beneficial, but this technique can cause severe surgical trauma. External fixation with stents does not properly correct and fix the displaced dorsal fracture fragments. Therefore, no current treatments are sufficient to adequately resolve these fractures, making them a challenge for orthopedic surgeons [5, 6].

In recent years, the introduction of the DVR anatomical palmar plate system has revolutionized treatment of distal radius fractures [7]. The DVR plate system is direct and easy to operate, allowing patients to quickly regain wrist movement [8]. The excellent biomechanical strength of the DVR plate is based on its ability to provide a three-dimensional scaffold structure and strong subchondral support for the articular surface of the distal radius. It locks the screw and screw structure by the fixed angle formed by the cross arrangement of two rows of screws at its far end. The role of these screws is to stabilize the subchondral bone and reduce disturbance to the dorsal tendon. The pull screw can fix the dorsally displaced fracture fragments and pull them to the radius, so as to resolve the fracture [9–11].

Although DVR plates are increasingly used in the treatment of distal radius fractures, it is difficult to fix a distal radius fracture with dorsal carpal displacement and free fragments by DVR anatomical volar plate alone. However, incorporating a dorsal incision to reduce the bone fragments with dorsal displacement would result in excessive surgical trauma. Therefore, we innovatively employ the volar approach to separate the distal radius and turn it to the volar side. After this, we could directly observe the displaced fracture on the dorsal side. Then, we use a Kirschner wire to temporarily fix the fracture block before further stabilizing and fixing it through the DVR plate. After these procedures, we could successfully repair the fracture of the distal radius with the displaced fracture block on the dorsal side.

The purpose of this study is to summarize the surgical method of the distal palmar approach combined with DVR plate fixation after radius turnover for the treatment of intra-articular distal radius fractures accompanied by dorsally displaced free fragments, as well as to evaluate the therapeutic effects of this technique.

## Methods

### General information

This study was approved by the Ethics Committee of Shenzhen People’s Hospital at Jinan University. All volunteers gave informed consent prior to participating in the study.

**Fig. 1:**
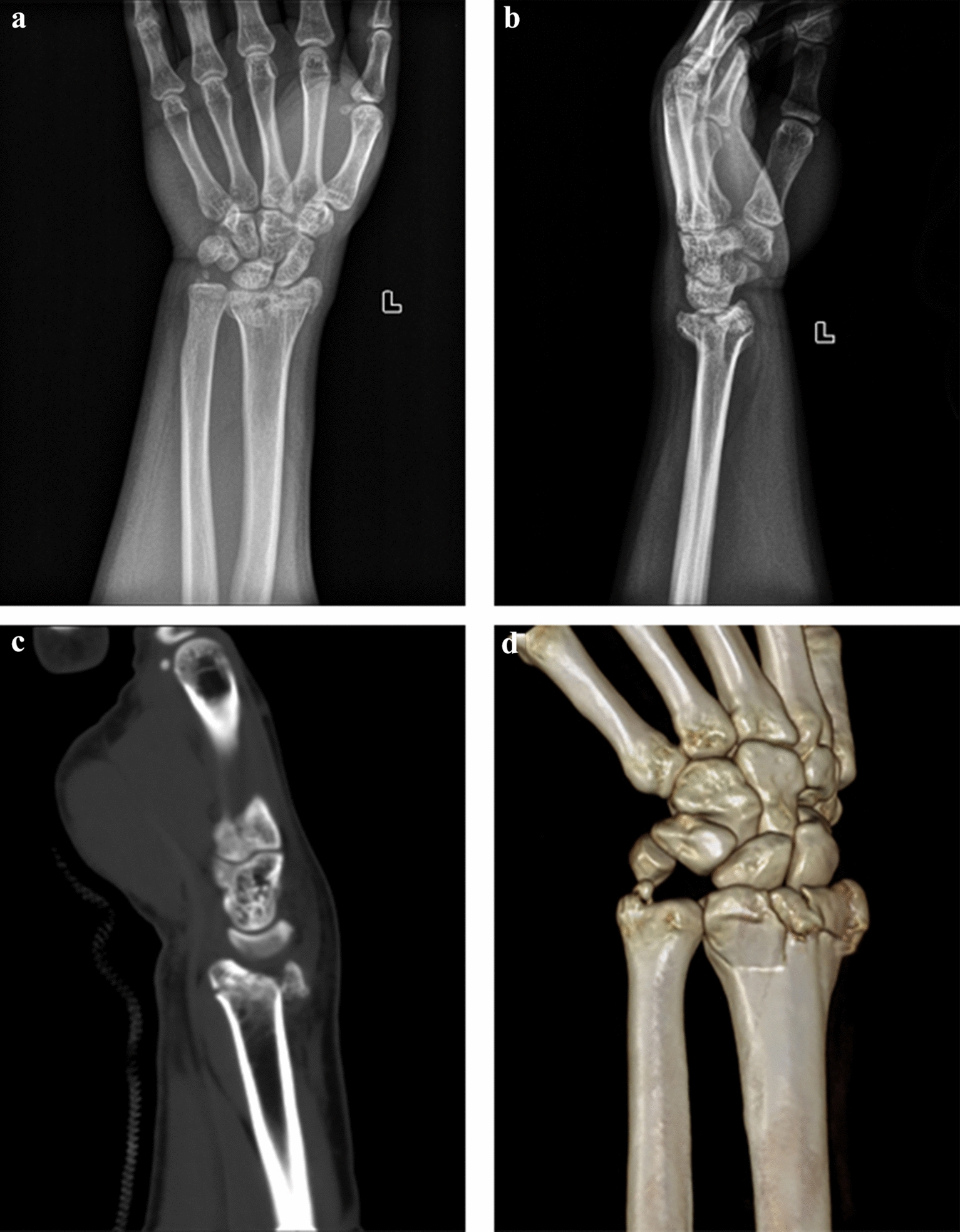
A 42-year-old man suffered from a comminuted fracture of the left distal radius after falling from a high place. A dorsal free fracture fragment is shown at the distal radius. **a**, **b** Anteroposterior and lateral X-rays before surgery. **c**, **d** Computed tomography of the fracture

From 2015 to 2019, 25 patients with intra-articular distal radius fractures accompanied by dorsally displaced free fragments were included in our study. Fifteen of the patients got their fractures from accidental fall, and 10 received their injuries from traffic accidents. All of the patients had closed fresh fractures. The study cohort included 14 males and 11 females with an average age of 34.5 years (21–50 years). According to AO classifications, all patients had type C fractures (Table 1), as determined by the presence of articular surface crushing, a fracture line that spread beyond the “watershed line” and the presence of dorsally displaced fracture fragments (Fig. 1). The patients were first treated with plaster external fixation and detumescence. The average operation time was 68.25 min (42–78 min), and the operation occurred an average of 4.5 days after injury (ranging from 0 to 7 days). The average follow-up time was 12.5 months (ranging from 8 to 22 months) (Table 2).

**Table 1:** Fracture type classification of the patients in our study by AO classification [12]

AO classification (2R3^−^)	Number of patients	
C 3.1	15	
C 3.2	8	Type C (complete articular)
C 3.3	2

**Table 2:** Demographics and clinical characteristics of patients

Variable	Value (mean value)
Patient	25
Sex (male: female)	14:11
Mean age (year)	34.5
Smoker	3
Time to surgery (day)	4.5
Operation time (min)	68.25
Follow-up period (months)	12.5

### Surgical technique

**Fig. 2:**
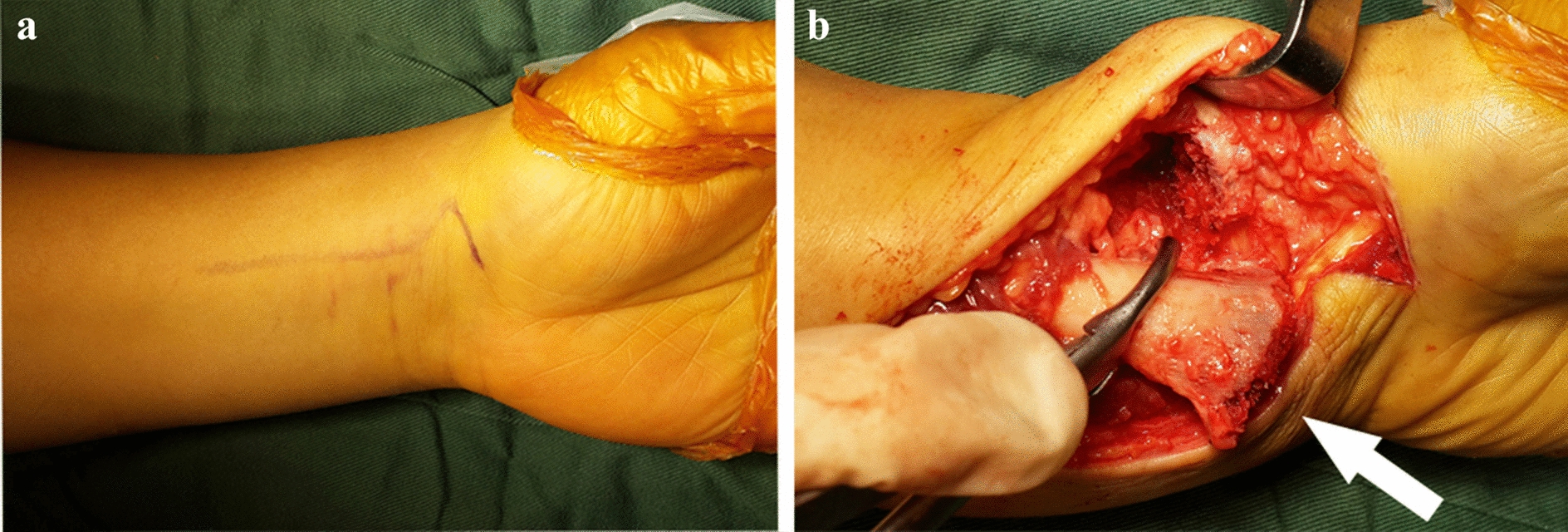
Incision and distal radius were reversed during the operation. **a** A zigzag incision was made across the wrist flexion crease to allow for better access and visualization. **b** Intra-focal exposure was obtained by pronating the proximal fragment out of the way with the turning radius (marked by the white arrow) using a bone clamp

After anesthesia, an 8 cm long incision was made along the flexor carpi radials (FCR). The skin, subcutaneous fascia and deep fascia were consecutively cut open, and the FCR was pulled toward the ulnar side. The median nerve was protected at the same time. The FCR was moved distally until it reached the level of the scaphoid node to enlarge the space under the palmar tendon of the forearm and expose the pronator quadratus muscle. After these manipulations, we were able to touch the distal radius and identify the palmar margin of the lunate fossa. We then marked both the “watershed line” and the proximal end of the lateral radius with sterile marker pen for later procedures. We used a sharp scalpel to cut both the proximal end of the lateral edge of the radius and the overlying ridge line, release the pronator muscle, cut off the middle support belt from the “watershed line”, peel off and expose the brachioradialis muscle stop, and obliquely disconnect it. Then, we turned the proximal segment of the radius inward, peeled and exposed the distal articular surface of the radius, restored the distal articular surface under direct vision and turned the proximal segment back after confirming satisfactory recovery of the articular surface. We placed the DVR (Zimmer-Biomet) plate on the palmar side. The distal end of the plate was aligned with the proximal side of the “watershed line”. A Kirschner pin was inserted into the medial Kirschner pin hole of the proximal row of pin holes to fix the distal bone block. The correct position of the plate was determined by C-arm fluoroscopy lateral film and oblique lateral film (inclined 20–25°). At this angle, the gram needle should be 2.0–3.0 mm below the articular cartilage. To stabilize the lunar fossa, a 2.0 mm drill bit was used to drill from the ulnar side through a disposable quick guide. The length of the near row of screws was obtained by reading the scale of the round surface on the sounder. After confirming the drilling depth, the quick guide was removed with a screwdriver. Then, we used the same screwdriver to insert screws of the correct length into all distal pin holes, and cortical screws were screwed into the remaining proximal screw holes. After pulling out the temporary fixing Kirschner wire, fracture recovery was satisfactory based on the fluoroscopy observation, and there were no obvious abnormalities in wrist joint movement. After hemostasis, the brachioradialis tendon and the pronator were sutured, and the incision was closed (Fig. 2). All operations were performed by an experienced orthopedic surgeon.

### Postoperative care

After anesthesia subsided, all patients began to stretch and flex their wrists slightly and move their elbows freely without bearing weight or participating in distance exercise or heavy physical activity of the affected limbs. Patients were suggested to be cautious about twisting exercises within 3 months of the operation.

### Radiological outcome

**Fig. 3:**
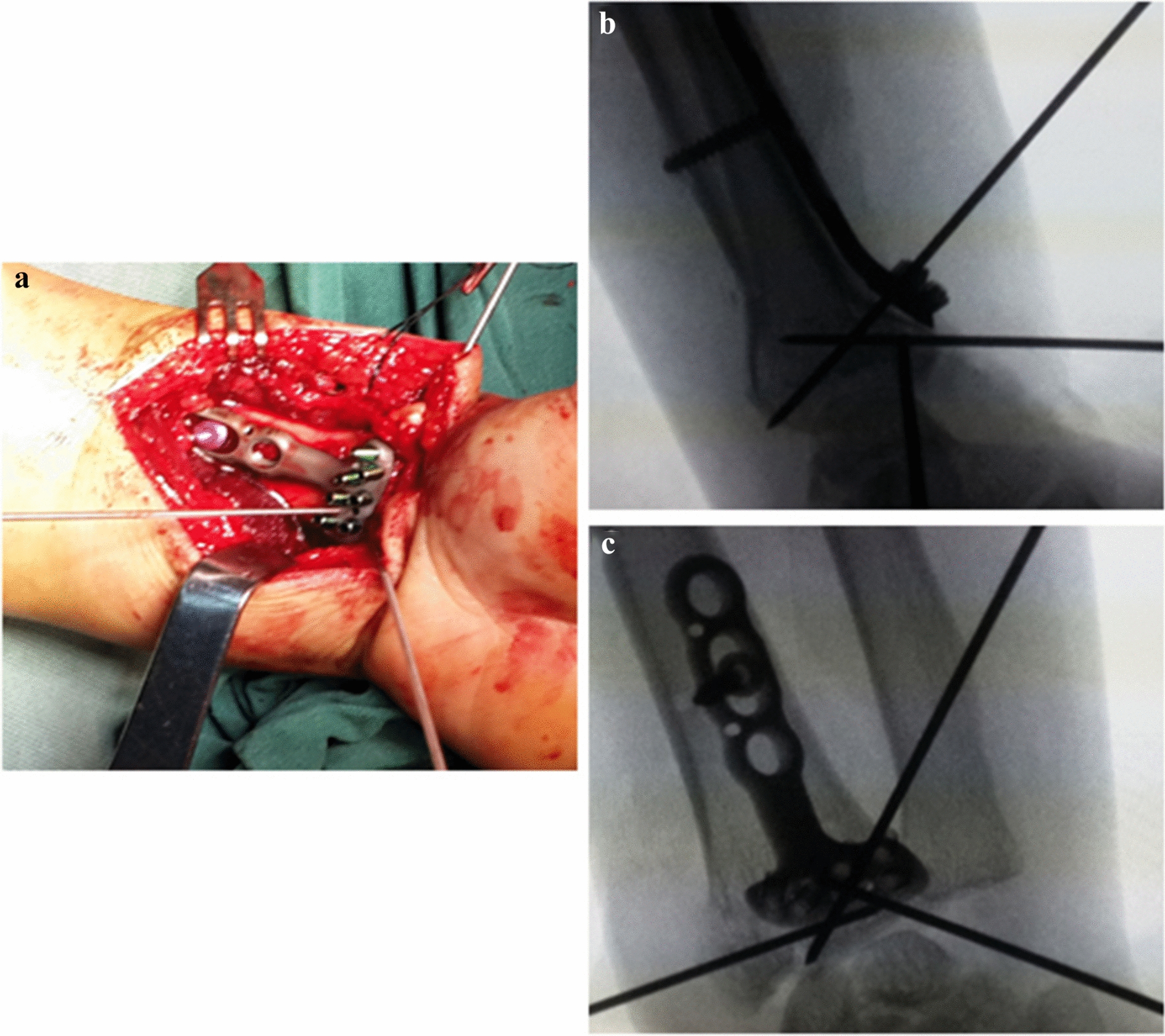
Image of the operation and fluoroscopy observation. **a** Intraoperative image after the placement of DVR with K-wires after open reduction of the fracture. **b**, **c** Fracture temporally fixed with two K-wires and plate temporally fixed with another K-wire (**b** lateral view; **c** anteroposterior view)

Preoperative X-ray and three-dimensional computed tomographic (CT) images were used to analyze the fracture types and observe the dorsally displaced fracture fragments far from the “watershed line”. X-ray films of anteroposterior and lateral carpal joints were taken immediately after the operation (Fig. 3).

**Fig. 4:**
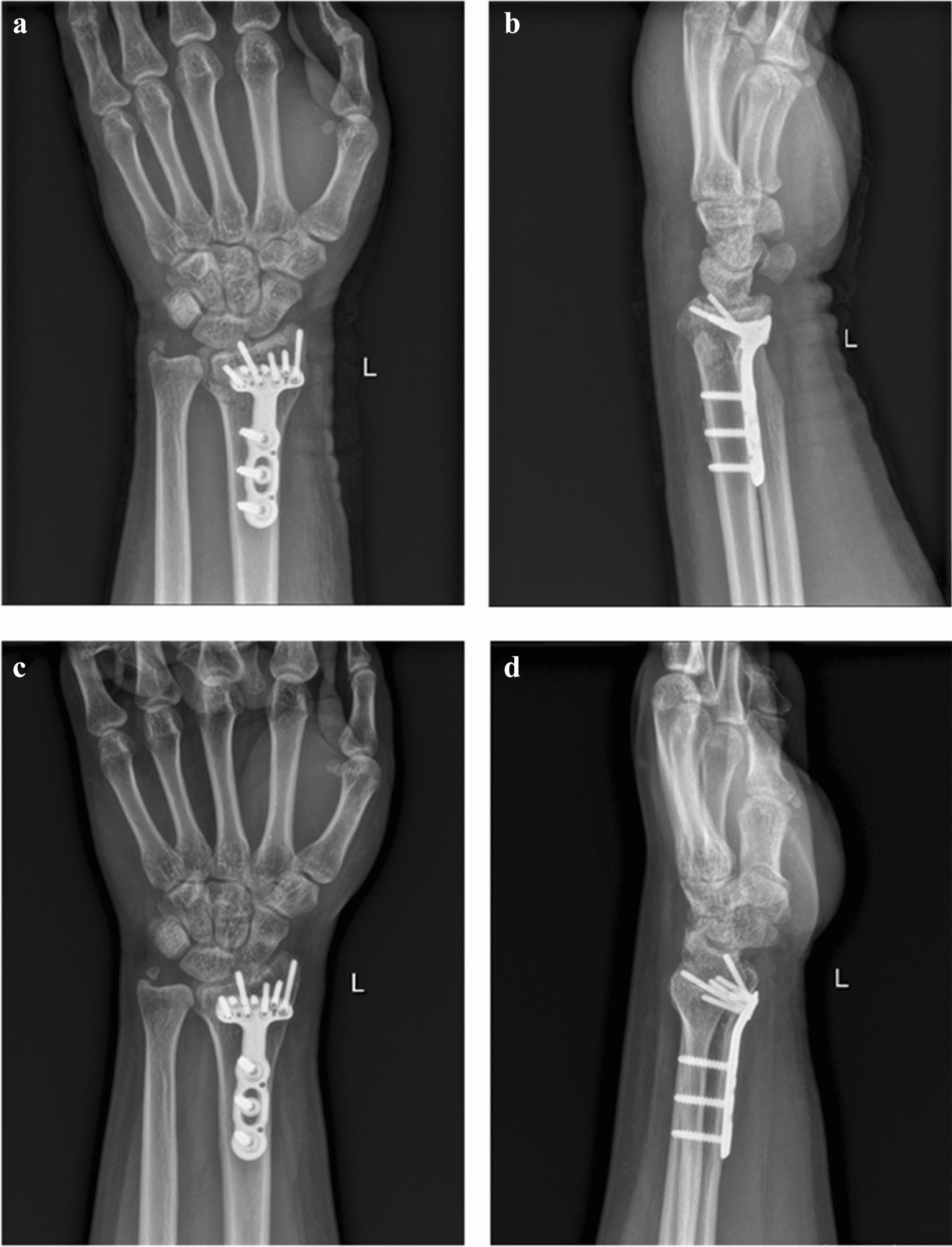
Radiographical images after the operation. **a**, **b** Anteroposterior (**a**) and lateral (**b**) radiographs showing that the alignment of the fracture and position of the plate were satisfactory on postoperative day 1. **c**, **d** Anteroposterior (**c**) and lateral (**d**) radiographs at 12-month post-operation, showing that the left wrist has completed bony union

At the end of the follow-up, X-ray was used to confirm fracture healing, distal radial height, volar tilt, and radial inclination, as well as to assess any evidence of traumatic arthritis (Fig. 4). Radiologic measurements were performed by two orthopedic doctors and a radiologist.

### Clinical evaluation

At the end of the follow-up, wrist function—including ROM, MMWs and DASH—was evaluated. ROM (dorsiflexion, volar flexion, ulnar deviation and radial deviation) of the wrist and forearm were measured with a goniometer. Pain visual analogue scale (VAS) was also evaluated.

### Complication

During the follow-up, we monitored possible postoperative complications, including wound infection, non-unions, malunions, tendon rupture, traumatic osteoarthritis, joint mobility disorders, persistent neuropathy and complex regional pain syndrome.

### Statistical analysis

Statistical analysis was performed using the SPSS statistical version 19.0 in this study. Data was shown as mean ± standard deviation (*x* ± *s*). The student *t* test was used for continuous variables. *P*-values less than 0.05 were regarded as statistically significant different.

## Results

All patients had satisfactory postoperative wound healing, and all patients showed evidence of bone healing during the follow-up. Functional recovery was satisfactory without any obvious complications. At the end of the follow-up, mean radial height was 10.5 mm (ranging from 8.1 to 12.6 mm), mean volar tilt angle was 9.28° (ranging from 5.7° to 12.8°) and mean radial inclination angle was 23.02° (ranging from 19.5° to 29.3°). There were no signs of post-traumatic arthritis based on patient radiography (Table 3; Fig. 4).

**Table 3:** Radiographic evaluation of the fracture after surgery

Variable	Mean (range)
Radial height (mm)	10.5 (8.1–12.6)
Volar tilt (°)	9.28 (5.7°–12.8°)
Radial inclination (°)	23.02 (19.5–29.3)

**Fig. 5:**
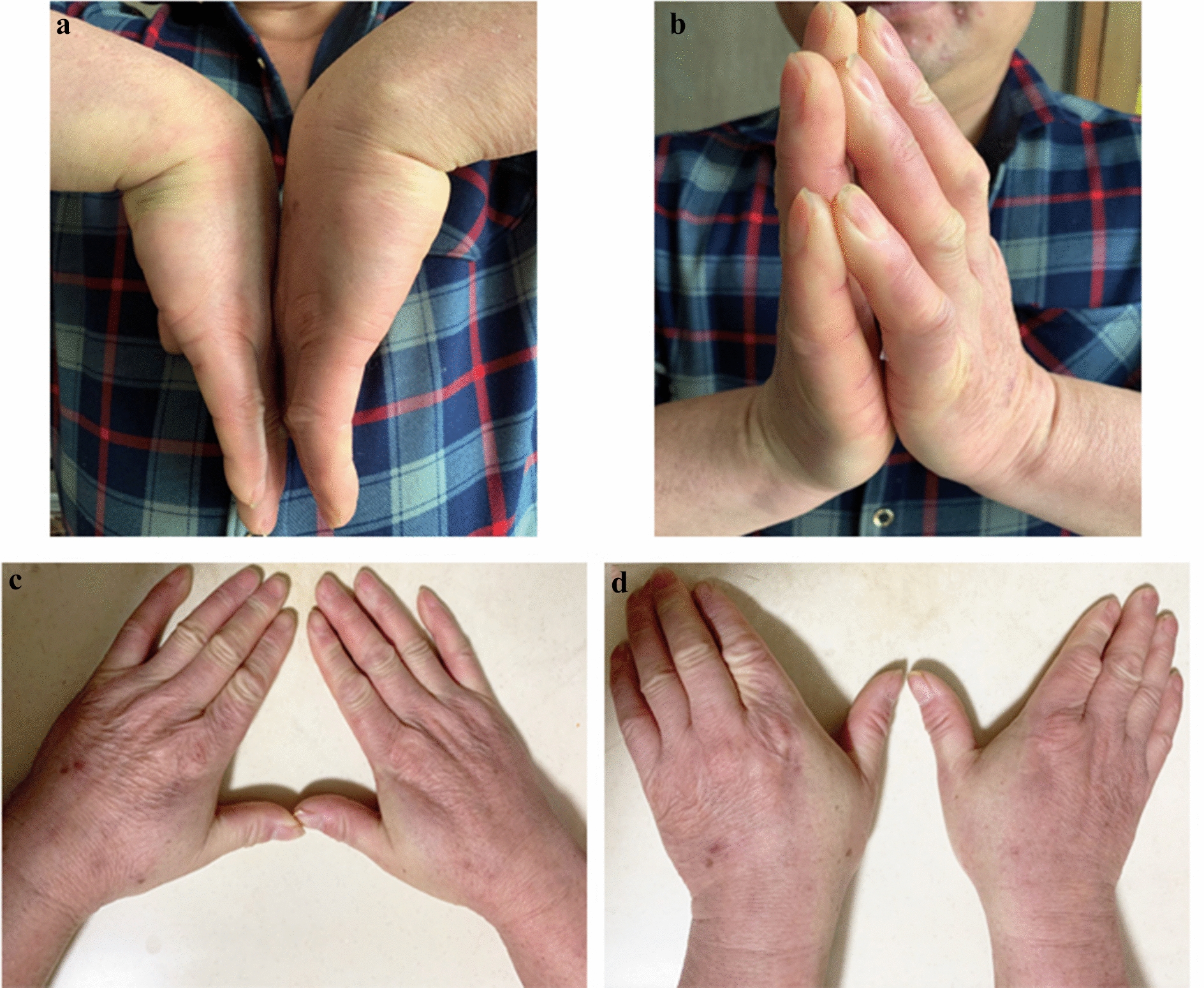
Excellent clinical results 12 months after surgery. The patient achieved volar flexion of 77° (**a**), dorsal flexion of 80° (**b**), radial deviation of 20° (**c**) and ulnar deviation of 23° (**d**)

All patients achieved satisfactory recovery and bony union. Wounds healed appropriately, and function recovered well without any obvious complications. At the end of the follow-up, the mean MMWS across all patients was 78.8° (ranging from 61° to 90°), and the mean DASH score was 16.25 (ranging from 11 to 21). The mean dorsiflexion ROM was 77.1° (ranging from 59° to 83°), and the mean volar flexion ROM was 76.5° (ranging from 62° to 81°). The average ROM of ulnar deviation angle was 21.4° (ranging from 15° to 28°), the average ROM of radial deviation angle was 17.5° (ranging from 12° to 23°) and the average VAS score of pain was 1.4 (ranging from 1 to 3) (Table 4 and Fig. 5).

**Table 4:** Clinical results

Score	Mean (range)
Dorsiflexion (°)	77.1 (59–83)
Volar flexion (°)	76.5 (62–81)
Ulnar deviation (°)	21.4 (15–28)
Radial deviation (°)	17.5 (12–23)
Modified Mayo wrist score	78.8 (61–90)
DASH score	16.25 (11–21)
VAS	1.4 (1–3)

## Discussion

It is difficult for orthopedic surgeons to deal with complex fractures, including intra-articular distal radius fractures accompanied by dorsal free fragments. Few studies for the treatment of this complex fracture have been reported, because a general volar locking plate cannot effectively fix the distal free fracture fragments [13, 14].

The anatomical volar locking plate system of the distal radius of DVR is a new internal fixation system that combines the advantages of previously existing internal fixation systems. First, the design of the plate is highly matched with the anatomic structure of the distal radius. The distal shape of the plate is well-matched to the “watershed line” and surface anatomical morphology of the distal radius on the volar side. Selvan et al*.* believe that DVR can reduce friction between the plate and tendon, more so than any other distal radius plate [15, 16]. When the plate is placed at the farthest end, it can effectively prevent tendon irritation, thus reducing disturbance to the soft tissue. Second, Vanhaecke et al. [16] believe that the key to this technology’s success is its ability to obtain solid fixation and perfect subchondral support. The DVR plate fits the “watershed line” of the distal radius on the volar side, providing strong support for volar marginal bone mass. The locking support rod and screw provide a solid nail/plate interface. The DVR system, consisting of two rows of 7 fixation screws and a patented three-dimensional scaffold generated by intersecting screws, has an improved supporting effect on the comminuted fracture and will not cause bone collapse. Third, the placement of a DVR plate is simple, leading to increased accuracy and decreased operation time. The DVR system is designed to use the “watershed line” on the palmar side of the distal radius as a natural anatomic mark and is placed by pushing the plate to the “watershed line”, resulting in an improved fit between the plate and bone surface and preventing the screw from entering the joint surface. Keeping screws out of the joint surface is one of the key steps for successful internal fixation.

Based on 187 cases with an average follow-up period of 2.5 years, Macfarlane et al*.* found that 8% of complications were caused by metacarpal plate screws, including three cases of tendon injury. This led them to recommended DVR for treatment of unstable distal radius fractures [17]. Two types of locking screws are used in the DVR system, threaded and unthreaded. Through mechanical experiments with a cadaver radius, Martineau et al. confirmed that there is no obvious difference between these two kinds of screws. However, they additionally concluded that the smooth rod screw is convenient to use and will not cause fracture block rotation [18]. Several reports have shown that the effect of DVR on anatomical structure and wrist function was satisfactory after the operation based on a 3-month follow-up [19, 20]. For fractures far from the distal radius watershed, many researchers and doctors tend to use an external fixation stent for fixation, as they believe that it is difficult to fix the plate effectively. However, Jorge Mora et al. [21] reported that the occurrence of complications from an external fixation stent is significantly higher than that from the DVR system.

With a single volar incision, it is difficult to reduce and fix the displaced fracture block with discontinuity against volar. To solve this problem, people have tried to reduce the displaced fracture block using manual traction or to add a dorsal incision to assist the reduction of the displaced fracture block. Traction does not correct the dorsal tilt of the distal fracture fragment. This is because the stout volar radiocarpal ligaments are shorter and they pull out to length before the thinner dorsal radiocarpal ligaments exert any traction [22–25]. In the present study, we cut off the brachioradialis muscle, turned the proximal radius over and completely exposed the distal dorsal fracture block and joint surface. Under this direct vision, we could recover the distal joint surface through first treating the dorsal joint surface, then the metacarpal joint surface, before finally turning the proximal radius over. Using these procedures, we could complete the operation on both sides of the metacarpal dorsal side with a single volar incision. After the operation, all wounds healed and wrist function was satisfactorily recovered.

However, we believe that not all distal radial fractures require radial turnover. In our opinion, the indications for radial turnover are as follows: cases with severe crushing of the articular surface; cases requiring dorsal fracture block reduction but presenting difficulties for dorsal incision. The contraindications for radial turnover are as follows: cases without the presence of serious joint surface crushing; cases with large distal metacarpal fractures. If the fracture on the articular surface is not serious, radius turnover is not required. In cases with large palmar fracture block, radius turnover is not worthwhile, as it is difficult to directly view the dorsal fracture block even after the turnover.

Our study has several limitations. First of all, as the chosen surgical approach is determined by both the type of fracture and the preference of the surgeon, this report reflects only the experiences of the surgeons in this study. Secondly, the case number in our study is small, and there is no control group. Compounded by the dearth of reports about the treatment of these complex fractures, we need more data to draw a solid conclusion. Moreover, we think that a longer follow-up period is needed to accurately evaluate the occurrence of traumatic osteoarthritis.

## Conclusions

For complex intra-articular distal radius fractures accompanying dorsally displaced free fragments, the distal palmar approach to the distal radius, with turning radius technology and DVR fixation, provides an excellent treatment option without dorsal trauma or obvious surgical complications. This surgical technique is simple and easy to employ, giving it great promise for clinical application.

## Data Availability

All the data and materials can be found in the manuscript.

## Abbreviations

DRFs: Distal radius fractures
DVR: Distal volaris radius
ROM: Range of motion
MMWS: Modified mayo wrist score
DASH: Disabilities of Arm, Shoulder and Hand
